# A Recessively Inherited Risk Locus on Chromosome 13q22-31 Conferring Susceptibility to Schizophrenia

**DOI:** 10.1093/schbul/sbaa161

**Published:** 2020-11-07

**Authors:** Tariq Mahmood, Mohammed E El-Asrag, James A Poulter, Alastair G Cardno, Anneka Tomlinson, Sophia Ahmed, Ahmed Al-Amri, Jamshid Nazari, Joanna Neill, Rifka S Chamali, Nancy Kiwan, Suhaila Ghuloum, Hamid A Alhaj, Juliette Randerson Moor, Shabana Khan, Hassen Al-Amin, Colin A Johnson, Peter Woodruff, Iain D Wilkinson, Manir Ali, Steven J Clapcote, Chris F Inglehearn

**Affiliations:** 1 Becklin Centre, Leeds and York Partnership NHS Foundation Trust, Leeds, UK; 2 Leeds Institute of Medical Research, University of Leeds, Leeds, UK; 3 Department of Zoology, Faculty of Science, Benha University, Benha, Egypt; 4 Division of Cardiovascular Sciences, School of Medicine, University of Manchester, Manchester, UK; 5 Leeds Institute of Health Sciences, University of Leeds, Leeds, UK; 6 Department of Psychiatry, University of Oxford, Oxford, UK; 7 NIHR-Sheffield Biomedical Research Centre, University of Sheffield, Sheffield, UK; 8 National Genetic Centre, Royal Hospital, Muscat, Oman; 9 Division of Pharmacy and Optometry, University of Manchester, Manchester, UK; 10 Weill Cornell Medicine-Qatar, Education City, Qatar Foundation, Doha, Qatar; 11 Psychiatry Department, Hamad Medical Corporation, Doha, Qatar; 12 Sheffield Health and Social Care NHS Foundation Trust, Sheffield, UK; 13 School of Biomedical Sciences, University of Leeds, Leeds, UK

**Keywords:** consanguineous/endophenotype/homozygosity/ chromosome 13q/risk haplotype

## Abstract

We report a consanguineous family in which schizophrenia segregates in a manner consistent with recessive inheritance of a rare, partial-penetrance susceptibility allele. From 4 marriages between 2 sets of siblings who are half first cousins, 6 offspring have diagnoses of psychotic disorder. Homozygosity mapping revealed a 6.1-Mb homozygous region on chromosome 13q22.2-31.1 shared by all affected individuals, containing 13 protein-coding genes. Microsatellite analysis confirmed homozygosity for the affected haplotype in 12 further apparently unaffected members of the family. Psychiatric reports suggested an endophenotype of milder psychiatric illness in 4 of these individuals. Exome and genome sequencing revealed no potentially pathogenic coding or structural variants within the risk haplotype. Filtering for noncoding variants with a minor allele frequency of <0.05 identified 17 variants predicted to have significant effects, the 2 most significant being within or adjacent to the *SCEL* gene. RNA sequencing of blood from an affected homozygote showed the upregulation of transcription from *NDFIP2* and *SCEL*. *NDFIP2* is highly expressed in brain, unlike *SCEL*, and is involved in determining T helper (Th) cell type 1 and Th2 phenotypes, which have previously been implicated with schizophrenia.

## Introduction

The substantial heritability of schizophrenia (60%–80%)^1–4^ is partly accounted for by common risk variants detected by genome-wide association studies (GWAS),^5,6^ rarer copy number variants (CNVs),^7,8^ and high penetrance alleles in genes such as *SETD1A*.^9,10^ Further ultra-rare variants could be shared by families^11,12^ or small, endogamous communities but would be missed in general population association studies because of their extremely low overall frequency.

Recessive alleles with a major effect on psychosis risk, if they exist, are likely to be enriched in affected individuals from populations with high consanguinity. This is because such progeny may have inherited 2 copies of the relevant variant (maternal and paternal) from a recent common ancestor.^13^ Evidence for such alleles comes from the observation that schizophrenia risk increases with consanguinity.^13–15^ Analysis of consanguineous families and cases with psychotic disorder has shown increased homozygosity^16^ and highlighted candidate genes and chromosomal regions of interest.^17–19^

The Pakistani population of West Yorkshire in the north of England comprised around 142 000 individuals in the 2011 census, the majority originating from Mirpur in Azad Kashmir, Pakistan. Around 37% of marriages in this community are between first cousins,^20^ resulting in unions that are both endogamous and consanguineous, with high background autozygosity (homozygosity-by-descent) and increased recessive disease due to enrichment for homozygosity of recessive alleles.^21^ There is an elevated prevalence of schizophrenia and other psychoses in the West Yorkshire Pakistani population.^22^ Like other migrant groups, they are subject to environmental risk factors such as low birth weight^23^ and risks associated with residence in deprived inner city areas.^24^ However, we hypothesized that part of the increase in risk could be due to recessive alleles of major effect predisposing to psychosis. We, therefore, looked within this community for consanguineous families with multiple cases of schizophrenia, which would support this assertion. Here, we report an analysis of one such family.

## Methods

A more detailed description of methods used in this study is included in the supplementary material. 

### Ascertainment and Diagnosis

Participants were recruited from the West Yorkshire Pakistani population, with ethical approval via Research Ethics Committee applications 08/H1313/17, 10/H1313/37, 11/H1310/1, 09/H1302/61, and 13/YH/0149 covering different aspects of the work. Clinical assessments were based on Schedules for Clinical Assessment in Neuropsychiatry (SCAN)^25^ or Positive and Negative Syndrome Scale (PANSS)^26^ interviews and review of case records.

### Homozygosity Mapping and Linkage Analysis

Homozygosity mapping was performed on Affymetrix 6.0 SNP (single nucleotide polymorphism) array data or SNP genotypes derived from whole exome sequencing (WES), using AgileMultiIdeogram (http://dna.leeds.ac.uk/agile/AgileMultiIdeogram/). Four chromosome 13q microsatellite markers were genotyped in 24 family members for whom DNA was available. Multipoint parametric linkage analysis was carried out using Superlink on-line (http://cbl-hap.cs.technion.ac.il/superlink-snp/). Nonparametric linkage was assessed using SimWalk 2.

### Whole Exome Sequencing

WES was performed using SureSelect Human All Exon V6 reagent (Agilent Technologies), with sequence data generated on a HiSeq 3000 (Illumina). Sequences were processed in SAM/BAM format using SAMtools^27^ and the Genome Analysis Toolkit (GATK).^28^ Synonymous variants, variants more than 2 base pairs (bp) beyond the splice junction, and those present in the single nucleotide polymorphism database (dbSNP) 146, the exome aggregation consortium (ExAC) v.0.3.1, or the genome aggregation database (gnomAD) v2.0 with a minor allele frequency (MAF) ≥5% were excluded. 

### Whole Genome Sequencing

Whole genome sequencing (WGS; 150-bp paired-end) was performed on an Illumina HiSeqX 10 sequencer (Edinburgh Genomics). Variants in the shared homozygous region were called in vcf format and filtered to exclude those with MAF ≥ 0.05 in gnomAD. Manual inspection of reads across the homozygous region was performed using the Integrative Genomics Viewer (IGV).^29^

### Transcriptional Analysis by RNA Sequencing

Peripheral blood was collected using PAXgene blood RNA tubes, and RNA extracted using a PAXgene RNA extraction kit, with quality confirmed on a Bioanalyzer (Agilent). Libraries were prepared using TruSeq RNA sample preparation Kit v2 and sequenced on a HiSeq3000 (Illumina). Genes were called as differentially expressed if *P*adj < .05 and they had an absolute log_2_ fold change (FC) of ≥1.

## Results

### Family Structure and Phenotype

**Fig. 1.**
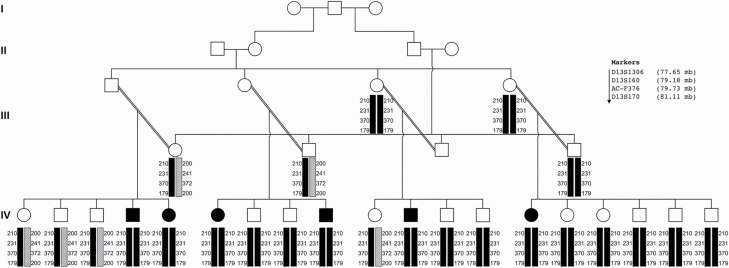
Pedigree and haplotype analysis. Four generation pedigree described in this study, with generation IV consisting of 4 nuclear families resulting from the marriage of half first cousins in generation III. Clear and shaded symbols denote unaffected and affected individuals, respectively. Double lines denote consanguinity. For those family members for whom DNA was available, a haplotype of 4 microsatellite markers on chromosome 13q22.3-31.1 is given below each individual, in centromeric to telomeric order. The risk haplotype is shaded black, while the only other haplotype observed is hatched.

The extended family studied (figure 1) consists of 4 nuclear families formed by intermarriages among 2 sets of siblings who are themselves first half cousins. Six family members have *Diagnostic and Statistical Manual of Mental Disorders*, 5th ed. (*DSM-5*)^30^ psychotic disorder diagnoses, based on consensus diagnoses of 2 psychiatrists: 5 have schizophrenia and 1 has other psychotic disorder, the latter with a history of delusions and manic symptoms. The onset of psychotic symptomatology occurred in the late teens or early twenties. Initially, affected individuals presented with auditory hallucinations, paranoid delusions, and formal thought disorder. Later, they developed negative symptoms and cognitive decline. Males showed exacerbation of symptoms after taking cannabis, but females did not take cannabis.

### Homozygosity Mapping

**Fig. 2.**
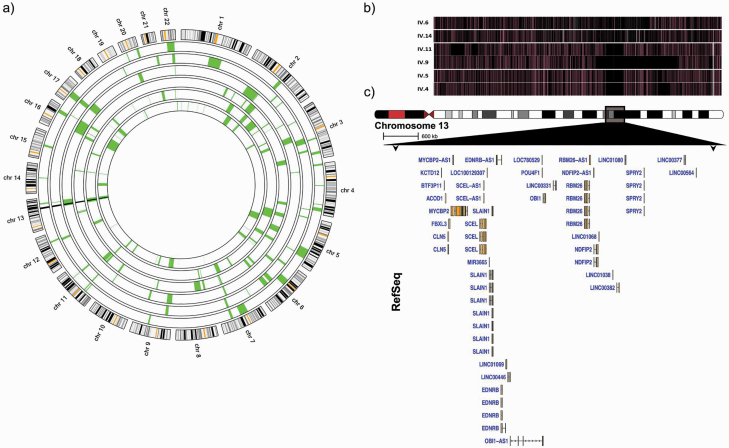
Homozygosity mapping. (a) Locations of homozygous regions identified in SNP or WES data from the 6 individuals with schizophrenia or “other psychotic disorder,” plotted against a circular ideogram of chromosomes 1–22 using the AgileMultiIdeogram software. SNP data for individuals IV4, 5, 9, and 11 and WES data for individual IV6 and 14, in order from the outer to the inner circles, are displayed as white circular bands in the center of the ideogram. Homozygous regions in each family member are shaded, while the homozygous region (Chr13: 76,483,001-82,585,713bp in hg19) shared by all family members is highlighted in black. (b) Homozygosity on chromosome 13q in the 6 affected individuals generated with the program SNPviewer. Each bar is a plot of the entire length of chromosome 13 in a single individual, with homozygous SNPs denoted by a black line and heterozygous SNPs by a lightly shaded line. The shared homozygous region appears as a black area shared by all 6 individuals. (c) 13q locus graphic displaying the genes and transcripts in the homozygous region.

Cytogenetic analysis of individual IV9 revealed a normal karyotype. Affymetrix 6.0 SNP genotypes were generated for IV4, IV5, IV9, and IV11, while SNP genotypes were extracted from WES of IV6 and IV14. Autozygosity mapping in these individuals (figure 2) revealed a single shared homozygous 6.1-Mb region on chromosome 13q. A homozygous region shared by chance across 4 sibships is highly unlikely. This observation, therefore, strongly suggests the segregation of a recessive, partial-penetrance risk allele. The region is bounded by SNPs rs17716584 (13q22.2) and rs7997648 (13q31.1) and contains 13 protein-coding genes: *KCTD12*, *ACOD1*, *CLN5*, *FBXL3*, *MYCBP2*, *SCEL*, *SLAIN1*, *EDNRB*, *POU4F1*, *RNF219*, *RBM26*, *NDFIP2*, and *SPRY2*. The Reference Sequence (RefSeq) database also documents 9 long intergenic nonprotein coding transcripts, 6 antisense transcripts, a microRNA, a pseudogene, and a putative gene (figure 2). The region also contains SNP rs9545047, which attained genome-wide significance in 1 schizophrenia GWAS^31^ and in the recent GWAS meta-analysis.^5^ Affected individuals are all homozygous for the at-risk A allele for SNP rs9545047.

Haplotypes of 4 microsatellite markers on chromosome 13q22.3-31.1 in family members are shown in figure 1. Allele frequency estimates from 27 unrelated Pakistanis suggest that the haplotype shared by affected individuals in the homozygous 13q region is rare in the Pakistani population (supplementary table S1). Analysis revealed that the 13q22.2-31.1 region is also homozygous in 12 apparently unaffected individuals in generations III and IV of the family. This suggests 33% (6/18) penetrance in homozygotes for the risk haplotype. Four siblings and 2 parents are heterozygous for the 13q risk haplotype and none are affected. A logarithm of the odds (LOD) score was calculated for linkage of the homozygous microsatellite haplotype with psychosis in the family using the allele frequencies in supplementary table S1. Parametric linkage analysis assuming a penetrance for the risk haplotype of zero in heterozygotes and 33% in homozygotes gave a LOD score of 3.02. Nonparametric linkage analysis gave a LOD score of >0.48 across all markers with a peak of 0.49 at D13S170.

### Further Phenotyping

Psychiatric screening of family members without psychotic disorders revealed 1 had anxiety symptoms, 2 had depression, and 1 had a history of self-harm. These 4 were all homozygous for the risk haplotype.

### Variant Screening by WES and WGS

WES in 3 affected homozygotes (IV6, IV9, and IV14) confirmed the shared homozygous region but revealed no potentially pathogenic protein-coding variants within it. WGS in IV11 revealed 5721 variants in the 6.1-Mb shared homozygous region. Excluding those with MAF > 0.05 in gnomAD and dbSNP146 and with a read depth of <2 reduced this to 183 variants (supplementary table S2). Seventeen of these variants were predicted to have a significant functional effect using the DeepSEA algorithm^32^ (supplementary table S3), including 2 with functional significance scores <0.01, 1 immediately upstream, and the other in an intron of the *SCEL* gene.

CNVs were neither detected by ExomeDepth^33^ nor observed via IGV within the shared homozygous region in WES from IV6, 9, and 14. No large structural variants were identified within the shared homozygous region in Manta analysis^34^ of WGS from IV11. Small indels identified in WGS were the same as or similar to variants present in the Database of Genomic Variation (DGV) and are, therefore, unlikely to be pathogenic. ExomeDepth analysis in WES from IV6, 9, and 14 also excluded large structural variants in other genomic regions reported to have genome-wide significance for association of CNVs with schizophrenia.^7^

### Transcriptome Analysis

RNA sequencing (RNA Seq) was carried out on cDNA from blood lymphocytes from a single affected individual (IV6) and 3 age, sex, and ethnically matched controls. Differential expression analysis using the package DSeq2^35^ revealed significant differences in the expression of 6 of the 13 genes in the shared homozygous region, with the upregulation of *NDFIP2* and *SCEL* as the most significant FCs observed (supplementary figures S2 and S3).

To determine whether SNPs within the homozygous region might be expression quantitative trait loci (e-QTLs) altering expression of a gene elsewhere, we examined the Gtex combined e-QTL track in the UCSC Genome Browser. Other than genes in the region, only *LMO7* and *UCHL3*, immediately proximal, were affected by e-QTLs in it, and neither were significantly altered in the above analysis. We then performed *cis-*eQTL analysis in genotype data from homozygotes with the GGtools Bioconductor package, but no linked *cis*-elements were discovered using a 5% false discovery rate threshold. 

## Discussion

Phenotypic, genetic, and transcriptomic analyses suggest that multiple cases of schizophrenia in an extended family are at least in part caused by a recessively inherited, partial-penetrance susceptibility allele or haplotype on chromosome 13q22-31. Support for the existence of a recessive schizophrenia risk locus in this family comes from the observation of affected individuals in each of 4 nuclear families formed by marriages between 2 sets of siblings who are first half cousins. This inheritance pattern is considered predictive of Mendelian recessive inheritance in other conditions.

However, apparent recessive inheritance in a single family could be coincidental in the context of this multifactorial disorder. We, therefore, sought further support for the existence of a partial-penetrance recessive schizophrenia risk locus in this family. This hypothesis predicts that all 6 affected family members, separated by 22 meioses from the common ancestor, will share a single homozygous region. Such a region would otherwise be highly unlikely given the complexity of the family structure and genetic distance between the individuals and sibships involved. Homozygosity mapping confirmed a single shared 6.1-Mb homozygous region on chromosome 13q22.2-31.1, between SNPs rs17716584 and rs7997648. This observation further supports the hypothesis of a high penetrance recessive locus, but quantification of its statistical significance is not possible due to the high endogamy and background homozygosity in the UK Pakistani population.^21^ Homozygosity mapping in a similar population, using WES data from 80 Qataris with schizophrenia (data not shown), revealed no enrichment for homozygosity at 13q22.3-31.1, suggesting that 13q homozygosity is not common in individuals with schizophrenia from other consanguineous populations.

Parametric linkage analysis between 13q markers and psychotic disorder in the family using a partial-penetrance recessive model gave a LOD score of 3.02, again supporting the hypothesis. Nonparametric linkage analysis gave a much lower score due to the lack of inferred data from the upper branches when analyzed without a recessive consanguineous model, but the pedigree shown in figure 1 strongly supports this model.

The 12 individuals homozygous for the 13q haplotype who do not have a psychotic disorder could be considered evidence against a 13q risk haplotype. However, other mental health problems were documented in 4 of the 12, pointing to a spectrum of severity in carriers for the risk haplotype. Homozygotes exhibited phenotypes ranging from being apparently unaffected (*n* = 8), through milder mental health problems (*n* = 4), to the most severely affected individuals who have psychosis (*n* = 6). This may indicate that the 13q risk haplotype acts in concert with environmental and other genetic factors, with cannabis use a possible contributory factor in male family members.^36^

There is additional support in the literature for a schizophrenia susceptibility locus on 13q22-31. A GWAS of schizophrenia in Ashkenazi Jews^31^ found an association with SNP rs9545047 (risk allele A global frequency 0.73), adjacent to the *RBM26* and *NDFIP2* genes within the shared homozygous region, which was confirmed in the latest GWAS meta-analysis.^5^ This is the only SNP currently associated with schizophrenia on chromosome 13. In addition, a GWAS in the Chinese population found that SNP rs2073831 in *KCTD12*, also within the shared homozygous region, was significantly associated with bipolar disorder (risk allele T global allele frequency 0.36).^37^ The homozygous 13q haplotype segregating in the family described includes the high-risk alleles for both of these SNPs, which may contribute to risk in the family. Linkage studies in multiplex families also provided evidence suggestive of linkage to this genomic region for both schizophrenia and bipolar disorder.^38–40^ Furthermore, a microdeletion encompassing the protein-coding genes *RBM26*, *NDFIP2*, and *SPRY2* was identified in a fetus with macrocephaly and macroglossia, suggesting a role for these genes in brain development.^41^

WES revealed neither rare protein-coding nor splice variants within this region. WGS identified 183 noncoding variants with MAF < 0.05, 17 of which are predicted to have a functional effect, the 2 most significant being within or adjacent to the *SCEL* gene. RNA seq in blood from an affected individual showed significant differences from controls in the expression of several genes in the interval, with the upregulation of transcription from the *SCEL* gene as the second most significant FC observed. *SCEL* encodes sciellin, not an obvious schizophrenia candidate gene as it is expressed almost exclusively in the skin, tongue, and tonsils, and functions in assembling the cornified envelope of mammalian keratinizing tissues. Furthermore, no significant skin abnormalities were noted in homozygotes for the risk haplotype.

*NDFIP2* was the most significantly upregulated transcript in blood from a homozygous affected individual. It encodes the NEDD4 family-interacting protein 2, involved in protein trafficking and ubiquitination. It is a stronger candidate for involvement in schizophrenia since it is highly expressed in the brain; lies within a haplotype block that includes schizophrenia-associated SNP rs9545047; was deleted in a fetus with brain abnormalities^42^; and is subject to rigorous constraint against missense or loss of function variants according to the gnomAD database. Furthermore, NDFIP2 is involved in the early differentiation of T helper (Th) cell subtypes Th1 and Th2^42^. Alterations of Th1-like cell-mediated and Th2-like antibody-related immune responses have been documented in schizophrenia and major depression.^43^

However, other genes in the region cannot be excluded, and several have well-defined roles in neuronal development and function.^44–47^ Furthermore, the present study is limited because transcriptional data are available only from blood RNA, there are a limited number of biological replicates available, and antipsychotic medication has a potential impact on transcription.^48^

In summary, we report a large family in which multiple individuals are affected with schizophrenia, giving the appearance of recessive inheritance of an allele of major effect. Homozygosity mapping identified a region of chromosome 13q shared by all affected individuals and by many apparently unaffected relatives. Further clinical examination revealed evidence for an endophenotype of milder psychiatric illness in several further homozygous individuals previously thought to be unaffected. No likely pathogenic-coding variants were identified, but transcriptomic analyses and other evidence highlight NDFIP2 as a strong candidate, possibly through its involvement in Th1 and Th2 differentiation.

## Funding

This work was supported by Medical Research Council UK project grant (MR/J004391/11 to S.J.C., C.F.I., A.G.C., and T.M.) and the Qatari National Research Fund (NPRP 7-1174-3-302 to H.A., C.F.I., S.G., A.G.C., M.A., and S.J.C) and benefitted from support from a Sir Jules Thorn Charitable Trust Award for Biomedical Research (JT/09 to C.A.J. and C.F.I.). A.G.C. was supported by Wellcome Trust Value In People award (473588).

## Supplementary Material

sbaa161_suppl_Supplementary_Figure_S1

sbaa161_suppl_Supplementary_Figure_S2

sbaa161_suppl_Supplementary_Table_1

sbaa161_suppl_Supplementary_Table_2

sbaa161_suppl_Supplementary_Material

## References

[1] Lichtenstein P , Yip BH, Björk C, et al. Common genetic determinants of schizophrenia and bipolar disorder in Swedish families: a population-based study. Lancet. 2009;373(9659):234–239. 19150704 10.1016/S0140-6736(09)60072-6 PMC3879718

[2] Cardno AG , Gottesman II. Twin studies of schizophrenia: from bow-and-arrow concordances to Star Wars Mx and functional genomics. Am J Med Genet. 2000;97(1):12–17. 10813800

[3] Hilker R , Helenius D, Fagerlund B, et al. Heritability of schizophrenia and schizophrenia spectrum based on the Nationwide Danish Twin Register. Biol Psychiatry. 2018;83(6):492–498. 28987712 10.1016/j.biopsych.2017.08.017

[4] Sullivan PF , Kendler KS, Neale MC. Schizophrenia as a complex trait: evidence from a meta-analysis of twin studies. Arch Gen Psychiatry. 2003;60(12):1187–1192. 14662550 10.1001/archpsyc.60.12.1187

[5] Pardiñas AF , Holmans P, Pocklington AJ, et al.; GERAD1 Consortium; CRESTAR Consortium. Common schizophrenia alleles are enriched in mutation-intolerant genes and in regions under strong background selection. Nat Genet. 2018;50(3):381–389. 29483656 10.1038/s41588-018-0059-2 PMC5918692

[6] Lee SH , DeCandia TR, Ripke S, et al.; Schizophrenia Psychiatric Genome-Wide Association Study Consortium (PGC-SCZ); International Schizophrenia Consortium (ISC); Molecular Genetics of Schizophrenia Collaboration (MGS). Estimating the proportion of variation in susceptibility to schizophrenia captured by common SNPs. Nat Genet. 2012;44(3):247–250. 22344220 10.1038/ng.1108 PMC3327879

[7] Marshall CR , Howrigan DP, Merico D, et al.; Psychosis Endophenotypes International Consortium; CNV and Schizophrenia Working Groups of the Psychiatric Genomics Consortium. Contribution of copy number variants to schizophrenia from a genome-wide study of 41,321 subjects. Nat Genet. 2017;49(1):27–35. 27869829 10.1038/ng.3725 PMC5737772

[8] Sullivan PF , Daly MJ, O’Donovan M. Genetic architectures of psychiatric disorders: the emerging picture and its implications. Nat Rev Genet. 2012;13(8):537–551. 22777127 10.1038/nrg3240 PMC4110909

[9] Takata A , Xu B, Ionita-Laza I, Roos JL, Gogos JA, Karayiorgou M. Loss-of-function variants in schizophrenia risk and SETD1A as a candidate susceptibility gene. Neuron 2014;82(4):773–780. 24853937 10.1016/j.neuron.2014.04.043 PMC4387883

[10] Singh T , Kurki MI, Curtis D, et al.; Swedish Schizophrenia Study; INTERVAL Study; DDD Study; UK10 K Consortium. Rare loss-of-function variants in SETD1A are associated with schizophrenia and developmental disorders. Nat Neurosci. 2016;19(4):571–577. 26974950 10.1038/nn.4267 PMC6689268

[11] Glahn DC , Nimgaonkar VL, Raventós H, et al. Rediscovering the value of families for psychiatric genetics research. Mol Psychiatry. 2019;24(4):523–535. 29955165 10.1038/s41380-018-0073-x PMC7028329

[12] Homann OR , Misura K, Lamas E, et al. Whole-genome sequencing in multiplex families with psychoses reveals mutations in the SHANK2 and SMARCA1 genes segregating with illness. Mol Psychiatry. 2016;21(12):1690–1695. 27001614 10.1038/mp.2016.24 PMC5033653

[13] Mansour H , Fathi W, Klei L, et al. Consanguinity and increased risk for schizophrenia in Egypt. Schizophr Res. 2010;120(1-3):108–112. 20435442 10.1016/j.schres.2010.03.026 PMC2900407

[14] Bener A , Dafeeah EE, Samson N. The impact of consanguinity on risk of schizophrenia. Psychopathology 2012;45(6):399–400. 22890479 10.1159/000338714

[15] Dobrusin M , Weitzman D, Levine J, et al. The rate of consanguineous marriages among parents of schizophrenic patients in the Arab Bedouin population in Southern Israel. World J Biol Psychiatry. 2009;10(4):334–336. 19921976 10.3109/15622970701849960

[16] Lencz T , Lambert C, DeRosse P, et al. Runs of homozygosity reveal highly penetrant recessive loci in schizophrenia. Proc Natl Acad Sci U S A. 2007;104(50):19942–19947. 18077426 10.1073/pnas.0710021104 PMC2148402

[17] Knight HM , Maclean A, Irfan M, et al. Homozygosity mapping in a family presenting with schizophrenia, epilepsy and hearing impairment. Eur J Hum Genet. 2008;16(6):750–758. 18322454 10.1038/ejhg.2008.11

[18] Iqbal Z , Vandeweyer G, van der Voet M, et al. Homozygous and heterozygous disruptions of ANK3: at the crossroads of neurodevelopmental and psychiatric disorders. Hum Mol Genet. 2013;22(10):1960–1970. 23390136 10.1093/hmg/ddt043

[19] Kurotaki N , Tasaki S, Mishima H, et al. Identification of novel schizophrenia loci by homozygosity mapping using DNA microarray analysis. PLoS One. 2011;6(5):e20589. 21655227 10.1371/journal.pone.0020589 PMC3105082

[20] Sheridan E , Wright J, Small N, et al. Risk factors for congenital anomaly in a multiethnic birth cohort: an analysis of the Born in Bradford study. Lancet. 2013;382(9901):1350–1359. 23830354 10.1016/S0140-6736(13)61132-0

[21] Woods CG , Cox J, Springell K, et al. Quantification of homozygosity in consanguineous individuals with autosomal recessive disease. Am J Hum Genet. 2006;78(5):889–896. 16642444 10.1086/503875 PMC1474039

[22] Saleem M , Brewin A, Ding C et al Risk of psychosis in Yorkshire South Asians. J Psychiatr Intensive Care 2019;15:117–121.

[23] Cannon M , Jones PB, Murray RM. Obstetric complications and schizophrenia: historical and meta-analytic review. Am J Psychiatry. 2002;159(7):1080–1092. 12091183 10.1176/appi.ajp.159.7.1080

[24] Pedersen CB , Mortensen PB. Evidence of a dose-response relationship between urbanicity during upbringing and schizophrenia risk. Arch Gen Psychiatry. 2001;58(11):1039–1046. 11695950 10.1001/archpsyc.58.11.1039

[25] Wing JK , Babor T, Brugha T, et al. SCAN. Schedules for Clinical Assessment in Neuropsychiatry. Arch Gen Psychiatry. 1990;47(6):589–593. 2190539 10.1001/archpsyc.1990.01810180089012

[26] Kay SR , Fiszbein A, Opler LA. The Positive and Negative Syndrome Scale (PANSS) for schizophrenia. Schizophr Bull. 1987;13(2):261–276. 3616518 10.1093/schbul/13.2.261

[27] Li H , Handsaker B, Wysoker A, et al.; 1000 Genome Project Data Processing Subgroup. The Sequence Alignment/Map format and SAMtools. Bioinformatics 2009;25(16):2078–2079. 19505943 10.1093/bioinformatics/btp352 PMC2723002

[28] DePristo MA , Banks E, Poplin R, et al. A framework for variation discovery and genotyping using next-generation DNA sequencing data. Nat Genet. 2011;43(5):491–498. 21478889 10.1038/ng.806 PMC3083463

[29] Thorvaldsdóttir H , Robinson JT, Mesirov JP. Integrative Genomics Viewer (IGV): high-performance genomics data visualization and exploration. Brief Bioinform. 2013;14(2):178–192. 22517427 10.1093/bib/bbs017 PMC3603213

[30] American Psychiatric Association. Diagnostic and Statistical Manual of Mental Disorders. 5th ed. Arlington, VA: American Psychiatric Association; 2013.

[31] Goes FS , McGrath J, Avramopoulos D, et al. Genome-wide association study of schizophrenia in Ashkenazi Jews. Am J Med Genet B Neuropsychiatr Genet. 2015;168(8):649–659. 26198764 10.1002/ajmg.b.32349

[32] Zhou J , Troyanskaya OG. Predicting effects of noncoding variants with deep learning-based sequence model. Nat Methods. 2015;12(10):931–934. 26301843 10.1038/nmeth.3547 PMC4768299

[33] Plagnol V , Curtis J, Epstein M, et al. A robust model for read count data in exome sequencing experiments and implications for copy number variant calling. Bioinformatics 2012;28(21):2747–2754. 22942019 10.1093/bioinformatics/bts526 PMC3476336

[34] Chen X , Schulz-Trieglaff O, Shaw R, et al. Manta: rapid detection of structural variants and indels for germline and cancer sequencing applications. Bioinformatics 2016;32(8):1220–1222. 26647377 10.1093/bioinformatics/btv710

[35] Love MI , Huber W, Anders S. Moderated estimation of fold change and dispersion for RNA-seq data with DESeq2. Genome Biol. 2014;15(12):550. 25516281 10.1186/s13059-014-0550-8 PMC4302049

[36] Brzozowska NI , de Tonnerre EJ, Li KM, et al. The differential binding of antipsychotic drugs to the ABC transporter P-glycoprotein predicts cannabinoid-antipsychotic drug interactions. Neuropsychopharmacology. 2017;42(11):2222–2231. 28272498 10.1038/npp.2017.50 PMC5603813

[37] Lee MT , Chen CH, Lee CS, et al. Genome-wide association study of bipolar I disorder in the Han Chinese population. Mol Psychiatry. 2011;16(5):548–556. 20386566 10.1038/mp.2010.43

[38] Blouin JL , Dombroski BA, Nath SK, et al. Schizophrenia susceptibility loci on chromosomes 13q32 and 8p21. Nat Genet. 1998;20(1):70–73. 9731535 10.1038/1734

[39] Brzustowicz LM , Honer WG, Chow EW, et al. Linkage of familial schizophrenia to chromosome 13q32. Am J Hum Genet. 1999;65(4):1096–1103. 10486329 10.1086/302579 PMC1288243

[40] Detera-Wadleigh SD , Badner JA, Berrettini WH, et al. A high-density genome scan detects evidence for a bipolar-disorder susceptibility locus on 13q32 and other potential loci on 1q32 and 18p11.2. Proc Natl Acad Sci U S A. 1999;96(10):5604–5609. 10318931 10.1073/pnas.96.10.5604 PMC21907

[41] Poreau B , Lin S, Bosson C, et al. 13q31.1 microdeletion: a prenatal case report with macrocephaly and macroglossia. Eur J Med Genet. 2015;58(10):526–530. 26365529 10.1016/j.ejmg.2015.09.003

[42] Lund RJ , Löytömäki M, Naumanen T, et al. Genome-wide identification of novel genes involved in early Th1 and Th2 cell differentiation. J Immunol. 2007;178(6):3648–3660. 17339462 10.4049/jimmunol.178.6.3648

[43] Schwarz MJ , Chiang S, Müller N, Ackenheil M. T-helper-1 and T-helper-2 responses in psychiatric disorders. Brain Behav Immun. 2001;15(4):340–370. 11782103 10.1006/brbi.2001.0647

[44] Li M , Milligan CJ, Wang H, et al. KCTD12 modulation of GABA(B) receptor function. Pharmacol Res Perspect. 2017;5(4):e00319. 28713569 10.1002/prp2.319 PMC5508304

[45] van der Vaart B , Franker MA, Kuijpers M, et al. Microtubule plus-end tracking proteins SLAIN1/2 and ch-TOG promote axonal development. J Neurosci. 2012;32(42):14722–14728. 23077057 10.1523/JNEUROSCI.1240-12.2012 PMC6621459

[46] Huang L , Hu F, Xie X, et al. Pou4f1 and pou4f2 are dispensable for the long-term survival of adult retinal ganglion cells in mice. PLoS One. 2014;9(4):e94173. 24736625 10.1371/journal.pone.0094173 PMC3988073

[47] Marvaldi L , Thongrong S, Kozłowska A, et al. Enhanced axon outgrowth and improved long-distance axon regeneration in sprouty2 deficient mice. Dev Neurobiol. 2015;75(3):217–231. 25104556 10.1002/dneu.22224

[48] Pillai A . Decreased expression of Sprouty2 in the dorsolateral prefrontal cortex in schizophrenia and bipolar disorder: a correlation with BDNF expression. PLoS One. 2008;3(3):e1784. 18335055 10.1371/journal.pone.0001784 PMC2262156

