# Supplementary figures and images for: A Recessively Inherited Risk Locus on Chromosome 13q22-31 Conferring Susceptibility to Schizophrenia
Source: Schizophr Bull. 2020 Nov 7;47(3):796–802. doi: 10.1093/schbul/sbaa161 (PMC8084434; doi:10.1093/schbul/sbaa161)

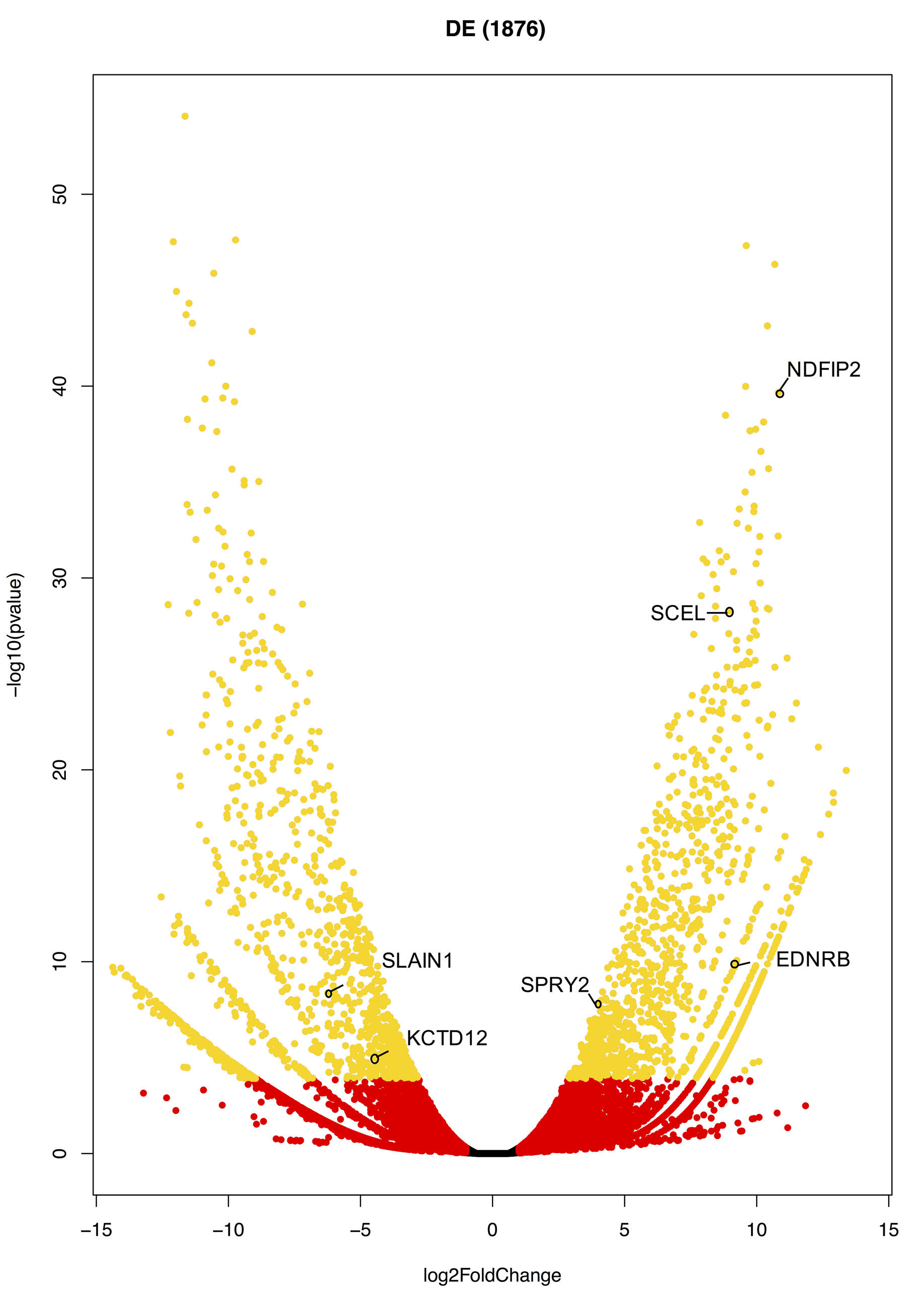

Supplement: sbaa161_suppl_Supplementary_Figure_S1 [file sbaa161_suppl_supplementary_figure_s1.png]

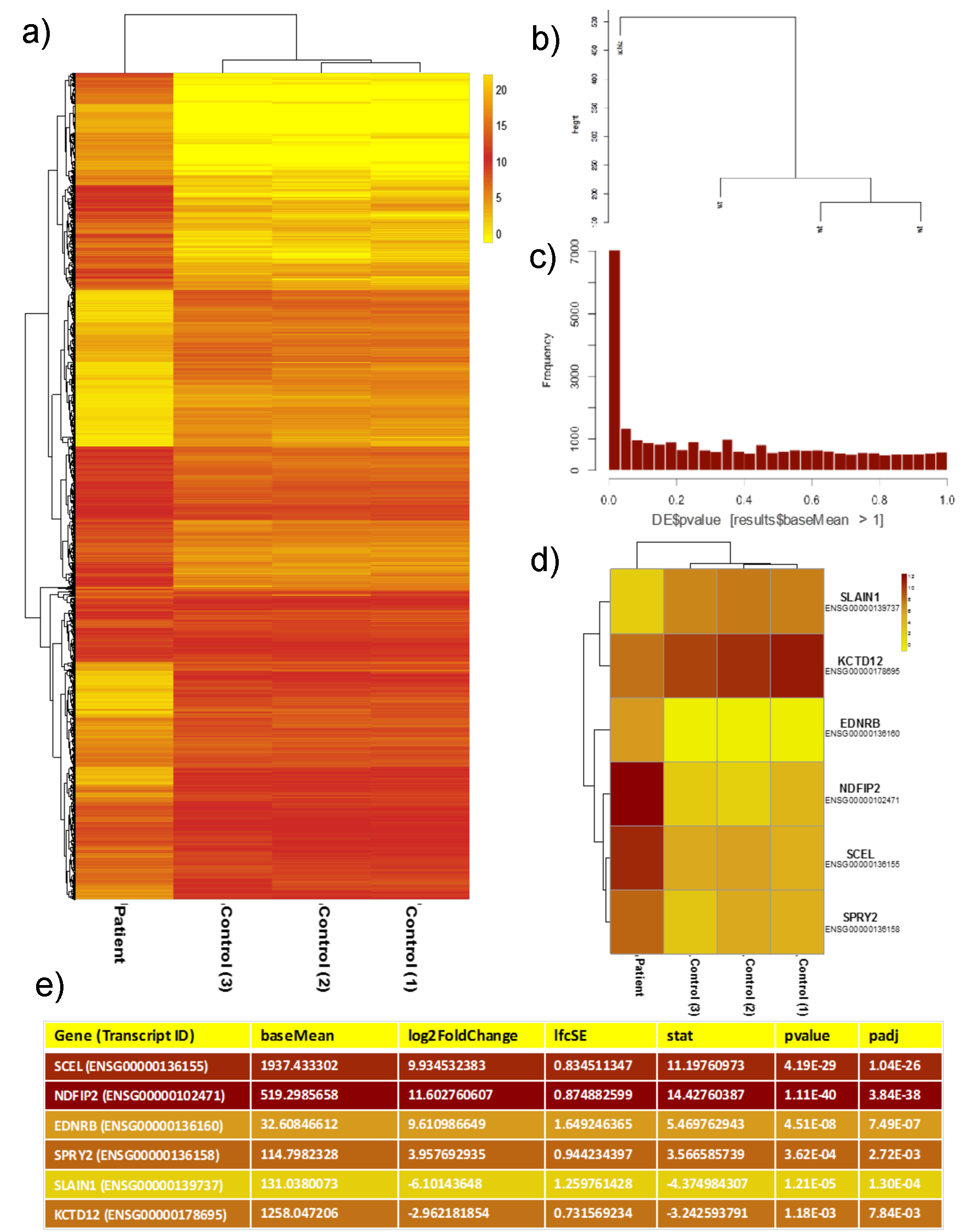

Supplement: sbaa161_suppl_Supplementary_Figure_S2 [file sbaa161_suppl_supplementary_figure_s2.png]
