# Supplementary material for: A Recessively Inherited Risk Locus on Chromosome 13q22-31 Conferring Susceptibility to Schizophrenia
Source: Schizophr Bull. 2020 Nov 7;47(3):796–802. doi: 10.1093/schbul/sbaa161 (PMC8084434; doi:10.1093/schbul/sbaa161)
Supplement: sbaa161_suppl_Supplementary_Material [file sbaa161_suppl_supplementary_material.doc]

**Supplementary material**

**Detailed methods**

*Diagnosis*

Clinical assessments were based on Schedules for Clinical Assessment in Neuropsychiatry (SCAN)^1^ or Positive and Negative Syndrome Scale (PANSS)^2^ interviews and review of case records.

*Homozygosity mapping and linkage analysis*

Homozygosity mapping was performed on Affymetrix 6.0 SNP array data or SNP genotypes derived from whole exome sequencing (WES), using AgileMultiIdeogram (http://dna.leeds.ac.uk/agile/AgileMultiIdeogram/). Chromosome 13q microsatellite markers D13S1306, D13S160 and D13S170 listed on the deCODE genetic map^3^, and AC dinucleotide repeat AC-F376 amplified using primers 5'-ccacgccagcctctattatt-3' and 5'-catgtgcagcactagccttg-3', were genotyped in 24 family members for whom DNA was available. Microsatellites were resolved on an ABI3130xl Genetic Analyser (Applied Biosystems). Multipoint parametric linkage analysis was carried out using Superlink on-line (http://cbl-hap.cs.technion.ac.il/superlink-snp/). Non-parametric linkage was assessed using SimWalk 2.

*Whole exome sequencing (WES)*

WES was performed using SureSelect Human All Exon V6 reagent (Agilent Technologies, Santa Clara, USA), with sequence data generated on a HiSeq 3000 sequencer (Illumina). Reads were aligned to hg19/GRCh37 using the Burrows-Wheeler Aligner (BWA)^4^. Read quality assessment and adapter trimming were performed on FastQ files using Trim Galore. Sequences were processed in SAM/BAM format using SAMtools^5^ and the Genome Analysis Toolkit (GATK)^6^. PCR duplicates were removed by Picard, and VCF files were annotated using the Variant Effect Predictor^7^. Synonymous variants, variants more than 2 base pairs (bp) beyond the splice junction and those present in dbSNP 146, ExAC v.0.3.1 or gnomAD v2.0 with a minor allele frequency (MAF) ≥5% were excluded. Binary alignment map (BAM) files from affected and control samples sequenced in the same lane were analysed using ExomeDepth^8^ to compare read depths, with CNVs prioritised by Bayes factor.

*Whole genome sequencing (WGS)*

150-bp paired-end WGS was performed on an Illumina HiSeqX 10 sequencer (Edinburgh Genomics, UK) to an average read depth of 32.95 (99.9% of bases covered by >10 reads). Reads were trimmed with CutAdapt, and fastq files were aligned to human genome sequence assembly hg19/GRCh37 with BWA^7^. Duplicate reads were removed and indels realigned according to GATK^6^ best practices. Variants in the shared homozygous region were called in vcf format and filtered to exclude those with MAF ≥0.05 in gnomAD. Those remaining were annotated using the Variant Effect Predictor^7^ and scored using the DeepSEA algorithm^9^. Analysis of the aligned BAM file with Manta^10^, using default settings, was performed to look for structural variants and indels. Manual inspection of reads across the homozygous region was performed using the Integrative Genomics Viewer (IGV)^11^.

*Transcriptional analysis by RNA sequencing (RNA-Seq)*

Peripheral blood was collected using PAXgene blood RNA tubes, and RNA extracted using a PAXgene RNA extraction kit, with quality confirmed on a Bioanalyzer (Agilent, Santa Clara, CA). Libraries were prepared using TruSeq RNA sample preparation Kit v2 and sequenced on a HiSeq3000 (Illumina). Read quality was assessed using the FastQC tool and bases with quality scores <20 were trimmed with Trim Galore v 0.4.4. RNA sequence reads were mapped to the GRCh38 human reference genome using STAR aligner version 2.5.3a with the --outSAMtype BAM SortedByCoordinate option^12^. Gene-level counts were generated from BAM files using the featureCounts^13^ function of Rsubread (v 1.26.1) in R, with the Homo_sapiens.GRCh38.91.gtf file (Ensembl). Count normalization and differential expression were performed using DESeq2^14^, with the Benjamini-Hochberg false discovery rate (FDR) method used to determine adjusted P-values (padj). Genes were called as differentially expressed if padj <0.05 and they had an absolute log_2_ fold change (FC) ≥1. Principal component analysis (PCA) was performed and sample-to-sample distances were computed using Euclidean distance. Heatmaps and volcano plots were made in R with ggplot2 (v 2.2.1), ggbeeswarm (v 0.6.0), pheatmap (v 1.0.8), and RColorBrewer (v 1.1.2).

Cis eQTL analysis was performed using the All.cis function in the GGtools Bioconductor package (https://bioconductor.org/packages/release/bioc/html/GGtools.html). Genotype data were retrieved from 6 individuals homozygous for the risk haplotype, while the expression data were retrieved from the DESeq2 results. eQTL analysis over the homozygous region plus the adjacent 100 kb identified no linked cis-elements using a 5% false discovery rate as a significance threshold.

**Supplementary Figure Legends**

**Supplementary Figure S1.** RNA-Seq differential expression analysis volcano plot. Volcano plot displaying 1876 genes differentially expressed in blood from a family member with schizophrenia by comparison with that of three age, sex and ethnically-matched controls. The vertical axis (y-axis) plots the log_10_ mean expression value (*P*-value), and the horizontal axis (x-axis) displays the log_2_ fold change value. Gold dots represent the significantly differential expressed transcripts (*P*-value (padj) <0.05). Red dots represent the transcripts whose expression levels did not reach statistical significance (*P*-value (padj) >0.05. Positive log_2_FoldChange means up-regulated in schizophrenia patient compared to controls. Negative log_2_FoldChange means down-regulated in schizophrenia patient compared to controls.

**Supplementary Figure S2.** RNA-Seq differential expression heatmap. (**a**) Heat map of all differentially expressed genes, with log_2_ fold changes indicated by colour on the adjacent scale. (**b**) Cluster dendrogram of sample-to-sample distance to overview the similarities and dissimilarities between the schizophrenia patient and controls (schiz = schizophrenia patient, wt = unaffected individual. (**c**) Histogram of *P*-values for genes with mean normalized count greater than 1. (**d**) Six differentially expressed genes in the shared region, with log_2_ fold changes indicated by colour on the adjacent scale. (**e**) DESeq2 output table for differentially represented genes in the shared homozygous region of dataset aligned with STAR. baseMean = the average of the normalized counts taken over all samples. log_2_FoldChange = log_2_ fold change between the schizophrenia patient and controls. lfcSE = standard error of the log_2_ fold change estimate. stat = Wald statistic. *P*-value = Wald test *P*-value. padj = Benjamini-Hochberg adjusted *P*-value. Positive log_2_FoldChange denotes up-regulation in schizophrenia patient compared to controls. Negative log_2_FoldChange denotes down-regulation in schizophrenia patient compared to controls.

**Supplementary Tables**

**Supplementary Table S1.** Frequencies of alleles for the chromosome 13q22.3-31.1 microsatellite markers used to generate the linked haplotype and for linkage analysis, derived from 27 unrelated Pakistani individuals.

(see Supplementary Table 1.xls)

**Supplementary Table S2.** Variants with MAF <0.05 identified within the shared homozygous region on chromosome 13q22-31 in WGS from individual IV11, after filtering on quality score and read depth >1.

(see Supplementary Table 2.xls)

**Supplementary Table S3.** Variants with MAF <0.05 identified within the shared homozygous region on chromosome 13q22-31 in WGS from individual IV11, after filtering on quality score, read depth >1 and functional significance score <0.05 using the DeepSEA algorithm.

| Position | rsID | Ref | Alt | Significance |
| --- | --- | --- | --- | --- |
| chr13:78108244 | rs146588134 | C | T | 0.0030423 |
|  |  |  |  |  |
| chr13:78167435 | rs374537294 | G | GTGCGCGCGCGTC | 0.0078084 |
|  |  |  |  |  |
| chr13:81187209 | rs73230768 | C | G | 0.012750 |
|  |  |  |  |  |
| chr13:79095370 | rs111828935 | C | G | 0.014153 |
|  |  |  |  |  |
| chr13:79353637 | rs748171853 | AT | A | 0.015341 |
|  |  |  |  |  |
| chr13:77403697 | N/A | TAAAAAA | T | 0.018454 |
|  |  |  |  |  |
| chr13:79355044 | rs141158331 | C | T | 0.021118 |
|  |  |  |  |  |
| chr13:78467333 | rs183448451 | G | T | 0.021787 |
|  |  |  |  |  |
| chr13:78772499 | N/A | T | A | 0.023660 |
|  |  |  |  |  |
| chr13:76936115 | rs140676016 | G | T | 0.026288 |
|  |  |  |  |  |
| chr13:77370575 | rs539961414 | G | C | 0.026386 |
|  |  |  |  |  |
| chr13:79605074 | rs527697755 | C | A | 0.030567 |
|  |  |  |  |  |
| chr13:80408884 | N/A | GTT | G | 0.034291 |
|  |  |  |  |  |
| chr13:76628403 | N/A | C | C(A)_31_ | 0.038071 |
|  |  |  |  |  |
| chr13:79078611 | rs111520033 | C | T | 0.040140 |
|  |  |  |  |  |
| chr13:79117769 | N/A | C | C(AT)_6_ | 0.042771 |
|  |  |  |  |  |
| chr13:78699685 | rs78923324 | C | T | 0.046084 |

**References**

1. Wing JK, Babor T, Brugha T, et al. SCAN. Schedules for Clinical Assessment in Neuropsychiatry. *Arch Gen Psychiatry*. 1990;47:589-593.
2. Kay SR, Fiszbein A, Opler LA. The positive and negative syndrome scale (PANSS) for schizophrenia. *Schizophr Bull*. 1987;13:261-276.
3. Kong A, Gudbjartsson DF, Sainz J, et al. A high-resolution recombination map of the human genome. *Nat Genet*. 2002;31:241-247.
4. Li H, Durbin R. Fast and accurate short read alignment with Burrows-Wheeler transform. *Bioinformatics*. 2009; 25: 1754-1760.
5. Li H, Handsaker B, Wysoker A, et al. 1000 Genome Project Data Processing Subgroup. The Sequence Alignment/Map format and SAMtools. *Bioinformatics*. 2009;25:2078-2079.
6. DePristo MA, Banks E, Poplin R, et al. A framework for variation discovery and genotyping using next-generation DNA sequencing data. *Nat Genet*. 2011;43:491-498.
7. McLaren W, Gil L, Hunt SE, et al. The Ensembl Variant Effect Predictor. *Genome Biol*. 2016;17:122.
8. Plagnol V, Curtis J, Epstein M, et al. A robust model for read count data in exome sequencing experiments and implications for copy number variant calling. *Bioinformatics*. 2012;28:2747-2754.
9. Zhou J, Troyanskaya OG. Predicting effects of noncoding variants with deep learning–based sequence model. *Nat Methods*. 2015;12:931-934.
10. Chen X, Schulz-Trieglaff O, Shaw R, et al. Manta: rapid detection of structural variants and indels for germline and cancer sequencing applications. *Bioinformatics*. 2016;32:1220-1222.
11. Thorvaldsdóttir H, Robinson JT, Mesirov JP. Integrative Genomics Viewer (IGV): high-performance genomics data visualization and exploration. *Brief Bioinform*. 2013;14:178-192.
12. Dobin A, Gingeras TR. Mapping RNA-seq Reads with STAR. *Curr Protoc Bioinformatics*. 2015; 51:11.14.1-11.14.19.
13. Liao Y, Smyth GK, Shi W. FeatureCounts: an efficient general purpose program for assigning sequence reads to genomic features. *Bioinformatics*. 2014;30:923-930.
14. Love MI, Huber W, Anders S. Moderated estimation of fold change and dispersion for RNA-seq data with DESeq2. *Genome Biol*. 2014;15:550.
